# Comparative analysis of the DNA methylation landscape in CD4, CD8, and B memory lineages

**DOI:** 10.1186/s13148-022-01399-0

**Published:** 2022-12-15

**Authors:** Ze Zhang, Rondi Butler, Devin C. Koestler, Shelby Bell-Glenn, Gayathri Warrier, Annette M. Molinaro, Brock C. Christensen, John K. Wiencke, Karl T. Kelsey, Lucas A. Salas

**Affiliations:** 1 Department of Epidemiology, Geisel School of Medicine at Dartmouth, Lebanon, NH USA; 2 Department of Epidemiology, Pathology and Laboratory Medicine, Brown University, Providence, RI USA; 3 Department of Biostatistics and Data Science, University of Kansas Cancer Center, Kansas City, KS USA; 4 Department of Neurosurgery, University of California, San Francisco, San Francisco, CA USA; 5 Department of Molecular and Systems Biology, Geisel School of Medicine at Dartmouth, Lebanon, NH USA

**Keywords:** Immune response, Immune activation, CD4 T cell, CD8 T cell, B cell, TEMRA, Central memory cell, Effector memory cell, DNA methylation

## Abstract

**Background:**

There is considerable evidence that epigenetic mechanisms and DNA methylation are critical drivers of immune cell lineage differentiation and activation. However, there has been limited coordinated investigation of common epigenetic pathways among cell lineages. Further, it remains unclear if long-lived memory cell subtypes differentiate distinctly by cell lineages.

**Results:**

We used the Illumina EPIC array to investigate the consistency of DNA methylation in B cell, CD4 T, and CD8 T naïve and memory cells states. In the process of naïve to memory activation across the three lineages, we identify considerable shared epigenetic regulation at the DNA level for immune memory generation. Further, in central to effector memory differentiation, our analyses revealed specific CpG dinucleotides and genes in CD4 T and CD8 T cells with DNA methylation changes. Finally, we identified unique DNA methylation patterns in terminally differentiated effector memory (TEMRA) CD8 T cells compared to other CD8 T memory cell subtypes.

**Conclusions:**

Our data suggest that epigenetic alterations are widespread and essential in generating human lymphocyte memory. Unique profiles are involved in methylation changes that accompany memory genesis in the three subtypes of lymphocytes.

**Supplementary Information:**

The online version contains supplementary material available at 10.1186/s13148-022-01399-0.

## Introduction

Cardinal features of adaptive immune memory are the generation of long-lived populations of self-renewing cells and a more rapid proliferative response to antigen re-exposure [1–3]. Driving memory generation in CD4 and CD8 T cells is the antigen-stimulated clonal expansion of short-lived effector cells followed by a contraction phase and emergence of central and effector memory compartments [1–4]. Memory T cells are maintained in a “poised” transcriptional state that facilitates rapid response to subsequent infection. CD4 memory cells have many diverse effector phenotypes, while CD8 memory cells exhibit a more limited range of these phenotypes [5–7]. Both cell types retain proliferative potential, although this is more robust in the CD8 T cells [5, 7].

For B cells, antigenic challenge leads to rapidly proliferating, short-lived, antibody-secreting plasma cells and germinal center B cells [3, 8]. Similar to effector T cells, the vast majority of these two cell types are eliminated through apoptosis. The surviving antigen-specific B cells comprise two separate memory compartments: the long-lived antibody-secreting plasma cell and a slowly proliferating self-renewing memory B cell.

Researchers have sought to define the genes and pathways individually responsible for the differentiation of each major lineage component in the genesis of immunologic memory [3, 9]. Recent works have established that epigenetic changes, including changes in DNA methylation, are among the crucial alterations that contribute to memory formation and storage in T and B lymphocytes [2, 8, 10–12]. However, few comparisons of the DNA methylation profile between lineages have been made to date. Parsimony may be evident in selecting the biological pathways for the genesis of immunologic memory. Thus, it is possible that there exist shared mechanisms of action or epigenetic states that play central roles in this form of phenotypic development within lymphoid tissues. In support of this idea, Luckey et al. compared mouse and human T and B cells and found a common signature of both up- and down-regulated transcripts shared between memory T cells, memory B cells, and long-term hematopoietic stem cells [13]. These observations suggested that a shared phenotype of self-renewal in the hematopoietic system is linked at the molecular level. Other researchers have reported that a common feature of memory lymphocytes in both T and B lineages is the elevated expression of transcription factors that could serve to enforce quiescence [14]. Here, we examined the DNA methylation alterations in human CD4, CD8, and B lymphocytes that were isolated from peripheral blood using surface markers widely accepted to mark different memory states with the goal of elucidating lineage-specific and overlapping epigenetic pathways in these disparate components of the immune response.

## Methods

### Isolated cells

The discovery dataset includes 6 B naïve (Bnv), 7 B memory (Bmem), 6 CD4 naïve (CD4nv), 6 CD4 central memory (CD4cm), 6 CD4 effector memory, 12 CD8 naïve (CD8nv), 5 CD8 central memory (CD8cm), 4 CD8 effector memory (CD8em), and 5 CD8 terminally differentiated effector memory (TEMRA) cell samples (Table 1).

**Table 1:** Discovery dataset baseline characteristics

Sample	N	Mean age (SD)	Male N (%)	Caucasian N (%)	African American N (%)	Hispanic N (%)	Unreported race N (%)
B naïve	6	38.2 (10.1)	3 (50.0)	3 (50.0)	2 (33.3)	1 (16.7)	0 (0)
B memory	7	32.0 (12.0)	4 (57.1)	5 (71.4)	1 (14.3)	0 (0)	1 (14.3)
CD4T naïve	6	29.3 (12.3)	6 (100)	6 (100)	0 (0)	0 (0)	0 (0)
CD4T central memory	6	54.7 (13.8)	5 (83.3)	0 (0)	0 (0)	0 (0)	6 (100)
CD4T effector memory	6	39.5 (17.1)	5 (83.3)	0 (0)	0 (0)	0 (0)	6 (100)
CD8T naïve	12	32.2 (11.1)	6 (50.0)	6 (50.0)	0 (0)	0 (0)	6 (50.0)
CD8T central memory	5	34.6 (2.6)	3 (60.0)	0 (0)	0 (0)	0 (0)	5 (100)
CD8T effector memory	4	36.0 (2.7)	2 (50.0)	0 (0)	0 (0)	0 (0)	4 (100)
CD8T TEMRA	5	37.4 (5.8)	3 (60.0)	0 (0)	0 (0)	0 (0)	5 (100)

Naïve and memory B cells (CD19+ CD27− and CD19+ CD27+, respectively) were obtained from StemExpress. Using magnetic-activated cell sorting (MACS) techniques, cells were isolated from healthy adult blood (harvested from leukopaks treated with ammonium chloride solution for RBC lysis). Leukocytes were separated into CD27 positive and negative populations, followed by selecting CD19+ cells using magnetic-activated cell sorting (MACS) techniques. StemExpress confirmed phenotypes by flow cytometric analysis, and purities ranged from 90 to 98%. All donors were unique for naïve and memory cell populations. Naïve B cell samples were collected from three females and three males, as were memory B cell samples.

Naïve CD4 cells (CD4+ CD45RA+ CD45RO−) were obtained from StemCell Technologies. Target cells were isolated from six healthy adult male donors (WBCs harvested from leukopaks treated with ammonium chloride solution for RBC lysis) by MACS (StemCell Technologies EasySep kit # 17555 for naïve CD4+ cell enrichment).

CD4 central and effector memory cells were isolated in-house (Brown University) using a combination of MACS and FACS (fluorescent-activated cell sorting). Briefly, leukoreduction filters (obtained from the Rhode Island Blood Center) were back-flushed, and PBMCs were collected from Ficoll gradients. Samples were enriched for CD4 memory cells using Miltenyi kit #130-094-302 to deplete the bulk of unwanted cells. The resulting CD4 memory enriched cells were fluorescently labeled and cell sorted into central (CD4+ CD8− CD45RA− CD45RO+ CD62L+ CD197+) and effector (CD4+ CD8− CD45RA− CD45RO+ CD62L− CD197−) subpopulations. Six donors represented each subpopulation; four donors yielded sufficient numbers of central and effector cells for downstream analysis; the remaining donors were unique. Central memory samples were comprised of five male and one female donor, while the effector cell samples consisted of four male and two female donors. The purity range was 92–95% for central and 92–97% for effector memory cell isolations.

Naïve CD8 samples were a combination of commercial and in-house isolations. The commercial samples were obtained from StemCell Technologies, similar to the description above for CD4, but using their EasySep kits for human CD8 naïve cells (Easysep kit #19258). The commercial donors were four males and two females; sample purity ranged between 85 and 89% by flow cytometric verification. Additional CD8 naïve were isolated in house, along with TEMRA, central, and effector CD8 subtypes. All cells were isolated using a combination of MACS and FACS techniques. Whole blood leukoreduction filters were obtained from donors between 31 and 46 years old. PBMCs were enriched for CD8+ using Miltenyi REAlease Microbead kit (# 130-117-036), followed by MACS separation of CD45RA positive and negative populations, prior to further separation by FACS sorting into CD8 T cell subpopulations. The CD8+ CD45RA+ enriched cells were sorted into CD8+ CD45RA+ CD45RO− CD62L+ CD197+ naïve (purity, 88–97% by FACS) and CD8+ CD45RA+ CD45RO− CD62L− CD197− TEMRA (purity, 84–88% by FACS) subtypes. The CD8+ CD45RA− MACS enriched cells were sorted into CD8+ CD45RO+ CD27+ CX3CR1− central memory and CD8+ CD45RO+ CD27+ CX3CR1++ effector memory populations (both populations were 86–95% pure by FACS assessment). Additional file 2: Table 1 shows the antibodies used for the in-house isolated cells.

All samples were obtained using Institutional Review Board-approved protocols.

### DNA methylation measurement, preprocessing, and quality control

DNA underwent sodium bisulfite conversion, followed by quality control of the converted sample by methylation-specific PCR and hybridization on the Illumina Infinium Methylation EPIC array platform. The data were processed using sesame v.1.16 and minfi version 1.34.0. Samples were background corrected using negative out-of-band (noob) and dye-bias nonlinear bleeding correction [15–17]. Probes were masked if their detection out-of-band array hybridization p values (pOOBAH) were > 0.05, if the probe was marked as potentially polymorphic or cross-reactive, if they were non-CpG probes or if the probes were tracking to the X or Y chromosomes [18, 19]. CpGs were further subset to autosomal loci, as were probes with internal SNPs near the 3’ end of the probe, probes with non-unique mapping to the bisulfite-converted genome, and probes with off-target hybridization due to partial overlap with non-unique elements [20]. The final data set contained 538,086 CpG loci for downstream statistical analysis of CpG-specific differential DNA methylation between naïve versus memory cell states.

### Differential methylation analysis

We conducted a series of comparative methylation analyses to discover the CpG loci that were differentially methylated between cell types. First, methylation beta values between naïve and memory cells were compared in B cell, CD4, and CD8 lineages. Furthermore, within the CD4 and CD8 memory compartments, we compared methylation beta values between central memory and effector memory cells. Finally, to depict distinct methylation patterns in the CD8 TEMRA cells, we compared methylation beta values in the TEMRA cells versus CD8 central and effector memory cells, respectively. Specifically, linear models were fit independently to each CpG, with methylation beta-values as the dependent variable and cell-state identity (e.g., naïve or memory) as the independent variable. Due to the high dimensionality of this study and relatively modest sample size, empirical Bayes-based variance estimates were obtained using limma [21]. The false discovery rate (FDR) was calculated to control for multiple comparisons. CpG loci were sorted by the absolute value of the coefficient estimated from linear models. The cutoff for significance was set at FDR < 0.05 and |Δ Beta|> 0.2. To search for common pathways, the top 100 differentially methylated loci ranked by |Δ Beta| were selected to compare overlapping CpGs identified between naive and memory subtypes across all three lineages, central and effector memory in the CD4 and CD8 lineage, TEMRA versus central and effector memory in the CD8 memory compartment. The list was also expanded to the top 1000 for an extensive search.

### Clustering and pathway analysis

Heatmaps were generated for each comparison using the top 20 significantly differentially methylated loci by |Δ Beta|> 0.2 across cell states. Samples in the heatmap are sorted by similarity using unsupervised hierarchical clustering. Metascape [22] with the Gene Ontology Biological Process database was used to conduct the pathway enrichment analyses with the shared genes identified between naïve and memory for each of CD4, CD8, and B cells, and central and effector memory in CD4 and CD8 lineage, respectively. eFORGE [23] with the CD34 T0 database was used to identify transcription factors enriched for overlapping differentially methylated CpGs (DMCs) between naïve and memory cells across CD4, CD8, and B cell lineages. To test memory generation related CpGs for genomic location enrichment among all tested CpGs (n = 538,086), the Illumina *HumanMethylationEPIC* annotation and UCSC Genome Browser *UCSC_hg19_refGene* files were used, while the relation of probes to CpG islands and enhancers was identified from the *HumanMethylationEPIC* annotation file. To define the genomic regions as promoters, introns, exons, or intergenic for each probe, the *annotateWithGeneParts* function from the R-package *genomation* and the *UCSC_hg19_refGene* file were used to map the regions to all CpG loci on the Illumina *HumanMethylationEPIC* array. If a probe mapped to more than a single genomic region, the probe was assigned preferentially with the order: promoters, exons, introns, and intergenic. Fisher’s exact tests were conducted to calculate odds ratios (ORs), p-values, and 95% confidence intervals for genomic region enrichment of differentially methylated CpGs between naïve and memory cells in B cell, CD4, and CD8 lineages, respectively. Gene sets that represent cell states and perturbations within the immune system, i.e., C7: immunologic signature gene sets, from the Molecular Signatures Database (MSigDB), were combined with the R package *missMethyl* to identify enriched immunologic signatures for each cell state comparison. The UCSC Genome Browser was used to extract genomic information for specific genes of interest profiled for methylation status across the cell types.

## Results

Methylation beta values between naïve and memory cells were first compared in B cell, CD4, and CD8 lineages. Differential methylation analyses identified extensive epigenetic changes, and the numbers of significantly hyper- and hypo-methylated CpGs at the cutoff FDR < 0.05 and |Δ Beta|> 0.2 are shown in Table 2. The most characteristic changes associated with memory generation involved a loss of methylation, although both loss and gain of DNA methylation were observed in each instance. In the B cell naïve to memory comparison, 91.4% of the significantly altered loci were hypomethylated in memory cells. A consistent preponderance of significant hypomethylation in memory cells compared with naive was observed for CD4 (88.4%) and CD8 (91.4%) comparisons. In effector memory cells compared with central memory cells, the majority of the DMCs were hypomethylated in effector memory cells for both CD4 (88.9%) and CD8 (81.2%) lineages. The complete output from the comparative regression models describing effect size and statistical significance between cell types was included in Additional file 3: Table 2.

**Table 2:** Number of significantly hyper- and hypo-methylated CpGs at the cutoff FDR < 0.05 and |Δ Beta|> 0.2

|ΔBeta|> 0.2, FDR < 0.05	Hypermethylated N (%)*	Hypomethylated N (%)^#^
Bnv versus Bmem	7997 (8.59)	85,078 (91.41)
CD4nv versus CD4cm	6906 (11.92)	51,010 (88.08)
CD8nv versus CD8cm	7807 (8.65)	82,454 (91.35)
CD4cm versus CD4em	378 (11.19)	2999 (88.81)
CD8cm versus CD8em	3053 (18.29)	13,639 (81.71)
TEMRA versus CD8cm	2496 (46.52)	2869 (53.48)
TEMRA versus CD8em	584 (11.89)	4326 (88.11)

*Hypermethylated in Bmem, CD4cm, CD8cm, CD4em, CD8em, CD8cm, CD8em by row

^#^Hypomethylated in Bmem, CD4cm, CD8cm, CD4em, CD8em, CD8cm, CD8em by row

**Fig. 1:**
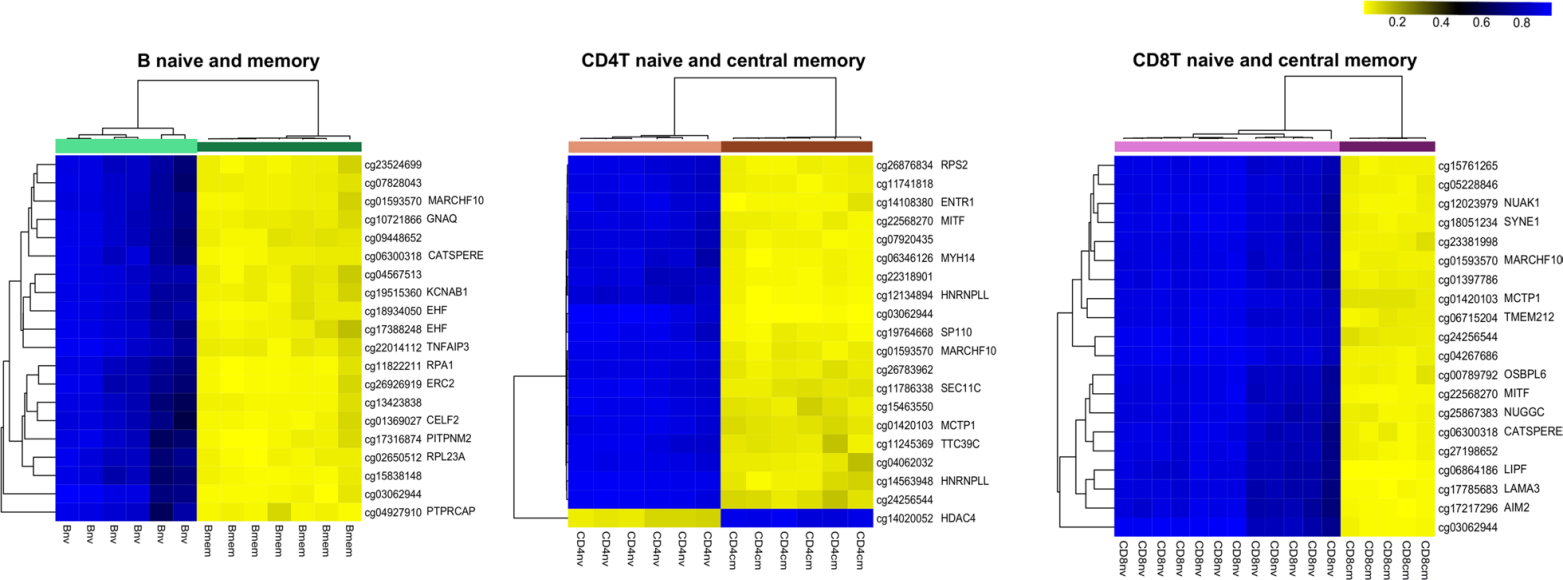
Top 20 significant differentially methylation CpGs between naïve and memory cells ranked by |Δ Beta| with their respective genes in B cell, CD4, and CD8 lineages

**Fig. 2:**
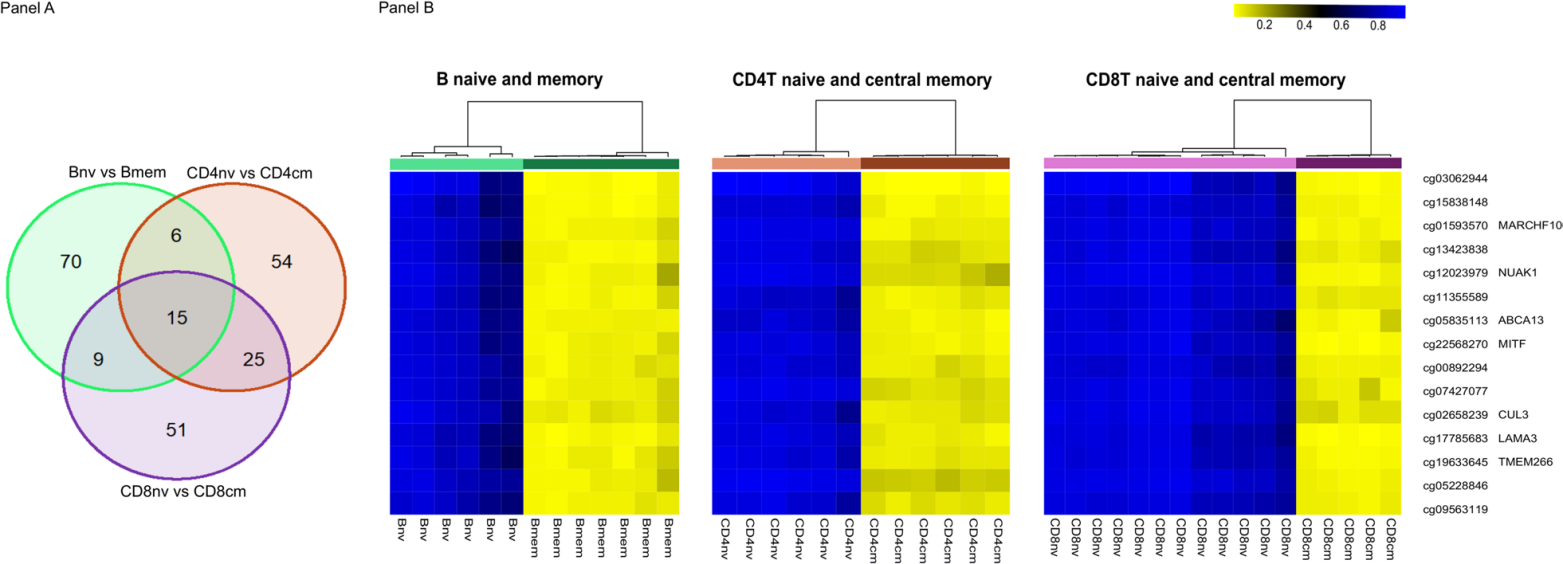
Shared differentially methylation CpGs between naïve and memory cells across B cell, CD4, and CD8 lineages. **a** Venn diagram demonstrating 15 out of 100 compared loci were in shared memory generation for all lineages. **b** Heatmap demonstrating methylation profile in 15 shared CpGs between naïve and memory cells across lineages

**Fig. 3:**
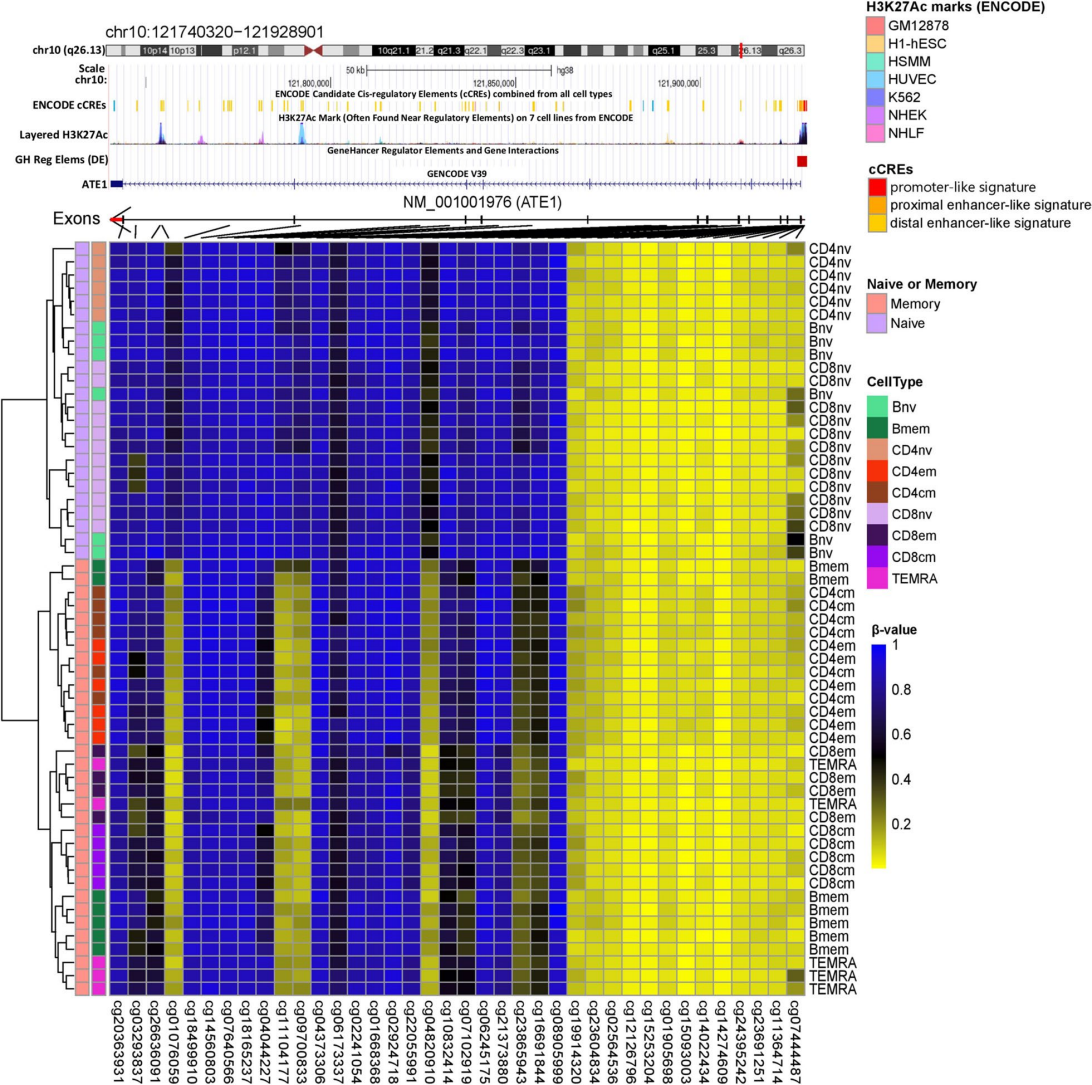
Heatmap of the methylation profile of the *ATE1* gene, demonstrating specific regions that are methylated in all subtypes of naïve cells but are demethylated in memory cells across B cell, CD4, and CD8 lineages

In memory activation, the top 20 significant DMCs ranked by |Δ Beta| with their respective genes were illustrated for B cell, CD4, and CD8 lineages in Fig. 1. The top 1000 DMCs, with their gene annotations, are included in Additional file 4: Table 3. Investigating the overlap of DMCs across three lineages identified 15 out of the top 100 DMCs by |ΔBeta| across all lineages (Fig. 2a), associated with seven genes (*MARCHF10, NUAK1, ABCA13, MITF, CUL3, LAMA3, TMEM266*) (Fig. 2b). To further explore the common pathways of memory generation across lineages, we expanded the DMC list to the top 1000 CpGs and identified 195 DMCs associated with 130 genes (Additional file 5: Table 4). All 195 CpGs were characterized by a loss of methylation during memory generation. Arginyltransferase 1 (*ATE1*) appeared with the most DMCs between the naïve and memory cells across the three cell lineages. *ATE1* was shown to be a key regulator in metabolism and relevant in pathogen response and inflammation as increased arginylation was often observed under these conditions [24]. A striking differential methylation pattern in ATE1 between naïve and memory cells was observed, with specific regions methylated in all subtypes of naïve cells but unmethylated in memory cells across B cell, CD4, and CD8 lineages (Fig. 3). The DMC sites common to T and B cells were found to be enriched in transcription factor binding sites, including homeobox and POU domain-containing factors (*POU6F1, POU1F1, OTX1*) and others implicated in developmental and immunologic processes (*BACH2, NR2F6, IRF3, IRF4, IRF5*), Supplemental Figure 1.

**Fig. 4:**
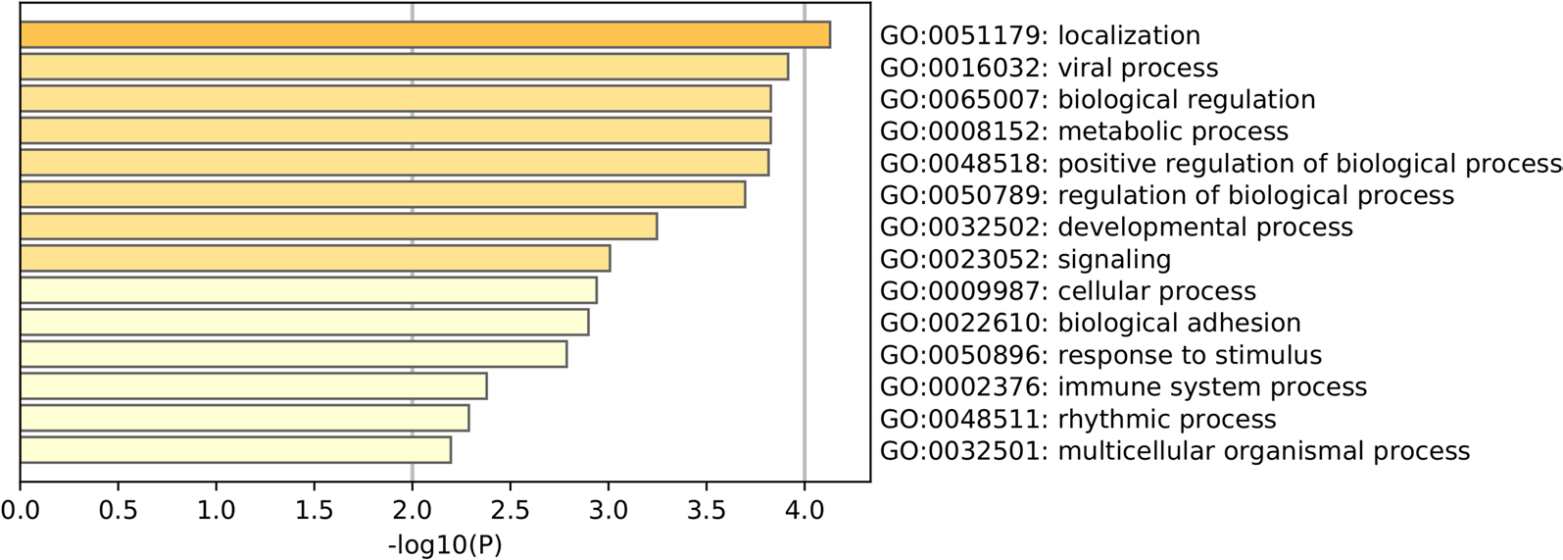
Significantly enriched GO biological processes with genes associated with shared differentially methylation CpGs between naïve and memory cells in the B cell, CD4, and CD8 lineages

We next conducted Gene Ontology (GO) enrichment analysis using Metascape [22] with the set of the 130 common genes observed for memory activation. The results identified biological processes that play critical roles in immune response, including viral process, response to stimulus, and immune system process (Fig. 4). Taken together, the results suggested existing epigenetic common pathways for memory activation across B cell, CD4, and CD8 lineages. Our data are additional evidence that DNA methylation plays an essential role in the lineage differentiation required for memory genesis.

**Fig. 5:**
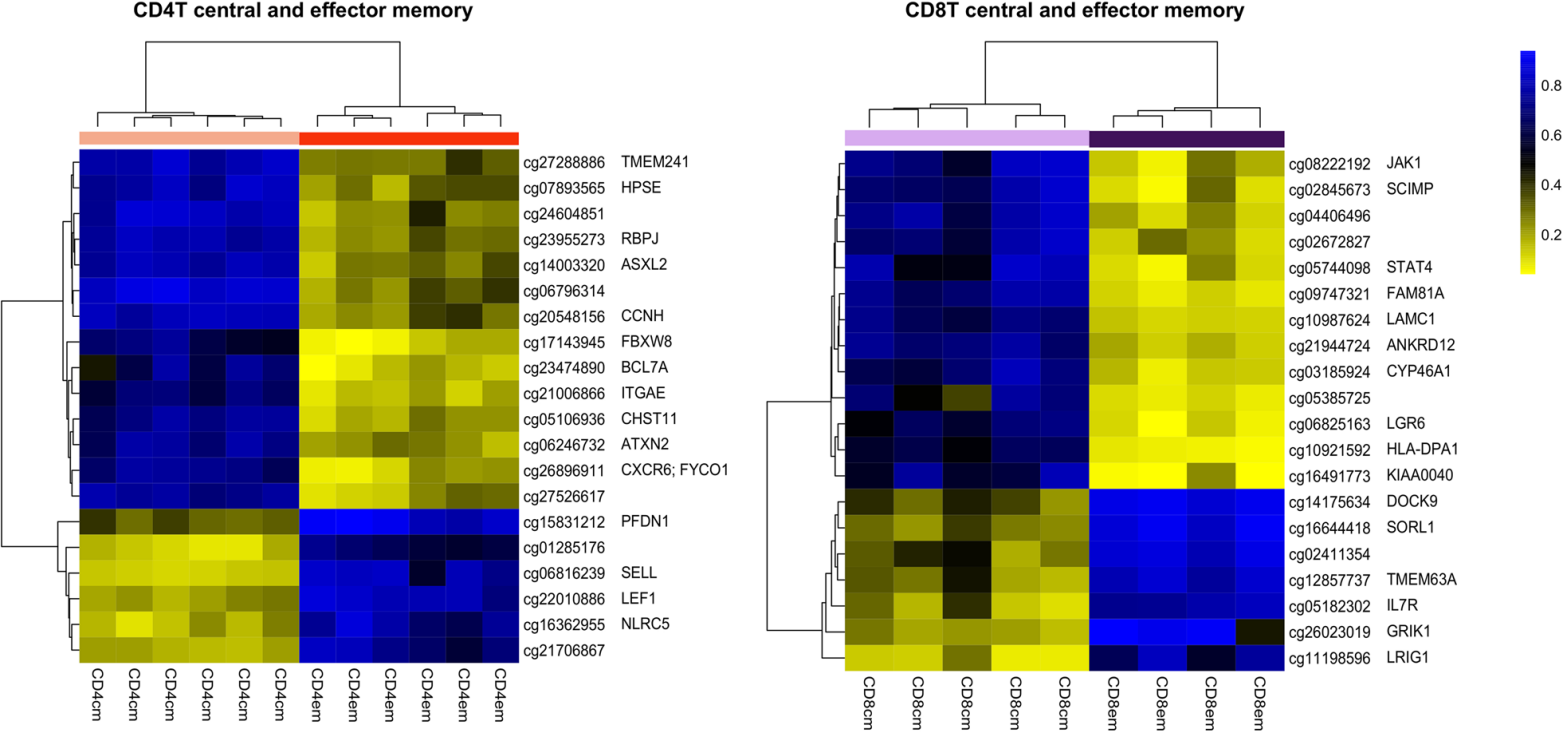
Top 20 significant differentially methylation CpGs between central and effector memory cells ranked by |Δ Beta| with their respective genes in the CD4 and CD8 lineages

Within the memory compartment, while numerous loci significantly marked different memory subtypes for both CD4 and CD8 lineages, the magnitude of the difference in methylation was qualitatively less than for memory generation. The top 20 significant DMCs ranked by |Δ Beta| between central and effector memory cells with associated genes are demonstrated in Fig. 5 for CD4 and CD8 lineage, respectively. The top 1000 DMCs with their respective genes can be found in Additional file 6: Table 5. Among the top 100 DMCs by |ΔBeta| between effector and central memory in both CD4 and CD8 lineages, we observed no shared CpGs. Among the top 1000 DMCs, 23 CpGs with 18 associated genes appeared to be shared during central to effector memory differentiation. However, the change of methylation direction was not consistent between CD4 and CD8 lineage (Additional file 7: Table 6). Metascape [22] was also used to identify enriched biological processes for central to effector memory differentiation. Similarly, stimulus and viral response, immune system process, and cell-level activity-related pathways were enriched for CD4 and CD8 central to effector memory differentiation (Additional file 1: Figure 2). The results indicated substantially unique epigenetic pathways leading to common biological pathways for CD4 and CD8 central to effector memory differentiation.

**Fig. 6:**
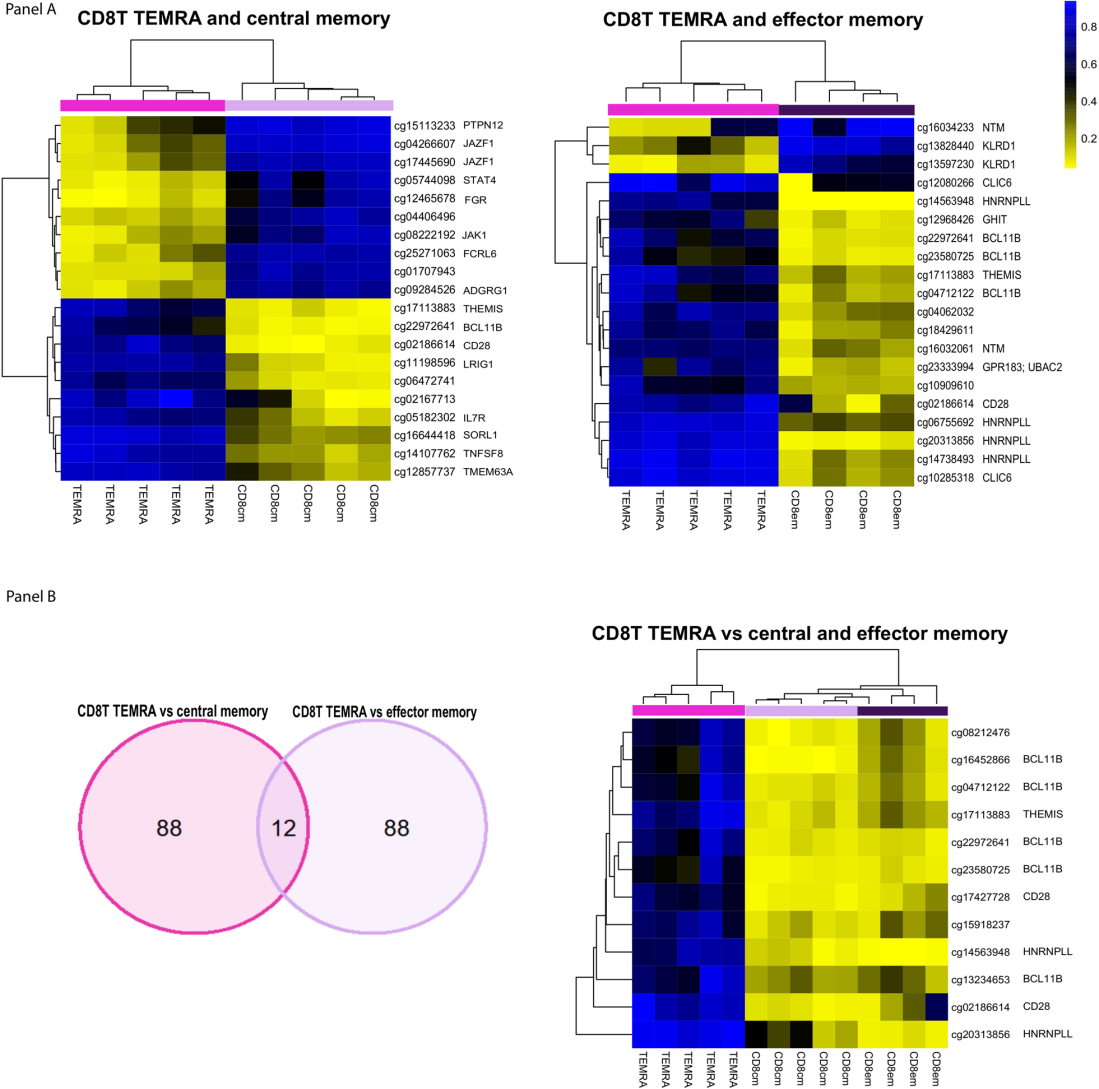
CD8 TEMRA methylation profiling compared to CD8 central and effector memory cells. **a** Top 20 significant differentially methylation CpGs in TEMRA versus central and TEMRA versus effector memory comparisons ranked by |Δ Beta| with their respective genes. **b** 12 shared differentially methylated loci between TEMRA versus central and TEMRA versus effector memory comparisons with their respective genes

**Fig. 7:**
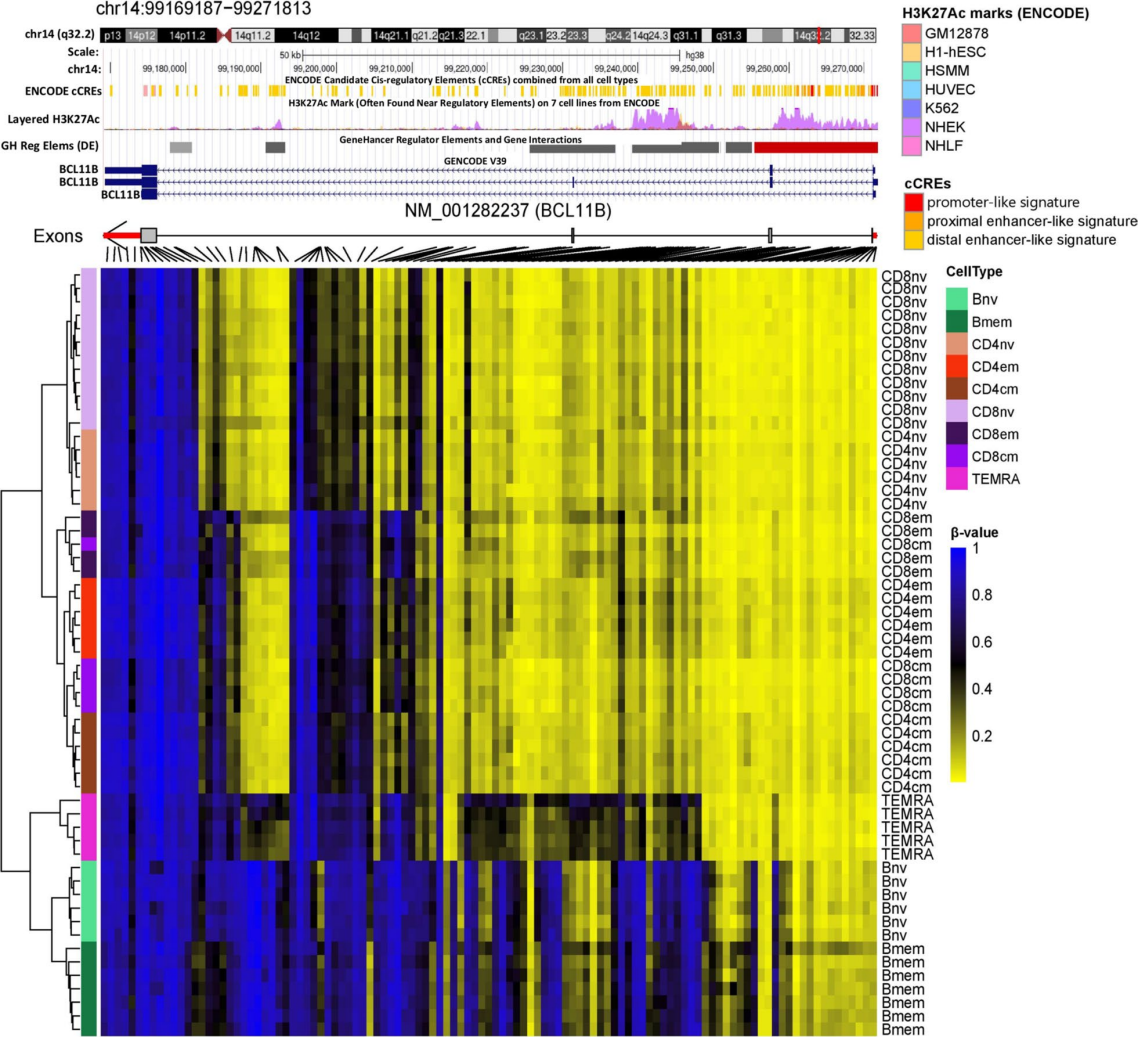
Heatmap of the methylation profile of the *BCL11B* gene, demonstrating unique methylation patterns in substantial regions in *BCL11B* for TEMRA compared to other cell types

CD8 terminally differentiated effector memory (TEMRA) cells are a subset of CD8 effector memory cells that expresses CD45RA that have distinct functions and localization than other memory cell subtypes [25]. We sought to delineate differential DNA methylation profiles for TEMRA compared to central and effector CD8 memory cell subtypes. The top 20 DMCs (and associated genes) for TEMRA versus CD8 central and effector memory cells are shown in Fig. 6a. The top 1000 DMCs with their respective genes can be found in Additional file 8: Table 7. By cross-checking the top 100 TEMRA DMCs between central and effector memory populations, we identified 12 overlapping CpGs (Fig. 6b). Among four genes (*BCL11B*, *THEMIS*, *CD28*, *HNRNPLL*) associated with these CpGs, *BCL11B* had the largest number of associated CpGs (5 out of 12, Fig. 6b). When expanding the comparative DMC number to 1000, 139 CpGs with 88 associated genes appeared to be shared between the CD8 TEMRA versus central memory and the TEMRA versus effector memory comparisons (Additional file 9: Table 8). Again, *BCL11B* had the most associated DMCs, demonstrating a decrease in methylation in 62 CpGs in TEMRA compared to central and effector memory cells (Additional file 9: Table 8). In previous research, *BCL11B* has been shown to be essential for multiple checkpoints during T cell development and, specifically, indispensable for effector CD8+ T cells[26, 27]. Our analysis revealed unique methylation patterns in substantial regions of the TEMRA *BCL11B* compared to other cell types (Fig. 7). Four striking differential methylation patterns were observed in *BCL11B,* i.e., B cell, CD4/CD8 naïve, CD4/CD8 central and effector memory, and TEMRA (Fig. 7).

Multiple genomic context enrichment analyses were conducted to investigate whether the memory generation-related CpGs are enriched in certain genomic locations. Consistently across B cell, CD4, and CD8 naïve to memory activation, increased methylation changes in the open sea were observed while CpG island and shore changes were highly under-represented (Additional file 1: Figure 2). Also, the intron and the intergenic regions were significantly enriched, whereas the promoter and the exon regions were under-represented for memory generation-related CpGs across all three lineages (Additional file 1: Figure 2). Additionally, the methylation changes during memory generation are highly associated with gene enhancer regions (Additional file 1: Figure 2). Our analyses revealed numerous loci in gene deserts that have not previously been associated with immunologic memory.

Lastly, the C7: immunological signature gene sets from the molecular signatures database (MSigDB) were used to explore potential immune cell-specific pathways that were enriched for the cell type DMCs identified by our comparative analyses. The top 10 enriched immunological signatures were illustrated for memory genesis in B cell, CD4, and CD8 lineage, respectively, in Additional file 1: Figure 3. Our results are concordant with those of gene expression studies using enrichment analyses we observed: 1) the gene set down-regulated in comparison of germinal center B cells versus naive B cells was enriched for hypermethylated CpGs in B memory generation, 2) the gene sets upregulated in comparison of CD4 naive T cells versus CD4 central memory T cells were enriched for hypermethylated CpGs in CD4 memory generation, and 3) the gene set down-regulated in comparison of naive CD8 T cells versus PD-1 low CD8 T cells was enriched for hypomethylated CpGs in CD8 memory generation. The complete list of the significantly enriched immunological signatures for each cell type comparison can be found in Additional file 10: Table 9.

## Discussion

We have investigated CpG loci altered by DNA methylation in putative human memory T and B cells and that are candidates in programming immune memory. There is now considerable evidence that the acquisition of both T cell [8, 28] and B cell memory [29] is, at least in part, epigenetically modulated.

Compared with CD4 naive cells, CD4 central memory cells tend to be less methylated at numerous loci. Consistently, Komori et al. [30], using a targeted bisulfite sequencing approach, reported 132 genes (corresponding to 466 CpGs) to have differential methylation between naïve and memory CD4 cells, finding 27% to have increased methylation and 73% to exhibit relative demethylation in memory compared with naïve CD4 cells. Notable among their findings was significantly less methylation at the *AIM2* and *CCL5* genes in memory CD4 cells, consistent with our findings and those of Nestor et al. [31]. Nestor et al. further show that both *AIM2* and *CCL5* undergo 5-hydroxymethylcytosine remodeling during the genesis of immune memory activation, something that cannot be distinguished from simple loss of methylation on the Illumina array [31]. In addition, several groups have reported [11, 32] that the *FOXP1* gene is epigenetically regulated in CD4 cells, being normally repressed in memory cells [33, 34]. Our data are consistent with this, showing a gain of methylation in the region previously shown to be repressive in memory cells, with concomitant loss of methylation in distinct memory CD4 cell regions likely representing known distinct isoforms. Similarly, it has long been known that transcriptional activation of the NF-kB pathway occurs rapidly after activation of CD4 memory cells [35], suggesting that memory in CD4 cells may be further regulated by additional loci in this extensive network, including loci that are NF-kB activation limiting and previously shown to be involved in the genesis of immune memory in CD4 cells, such as *NFATC2* [36]. The data herein support prior work showing *NFATC2* methylation to be directly involved in CD4 memory [30, 32], with over 30 loci showing loss of methylation in memory cells. Our data further supply numerous additional loci, which are excellent candidates for playing critical regulatory roles in generating immune memory in CD4 cell activation.

In the case of immune memory generation in the CD8 T cell lineage, there is considerable evidence that changes in DNA methylation also drive this process. For example, Rodriguez et al. [2] compared CD8 naïve and effector memory cells, reporting numerous loci exhibiting altered methylation. Our data are entirely consistent with Rodriguez et al. when comparing CD8 naïve and central memory cells, showing similar changes in methylation associated with loci coding for features such as cell adhesion (*ITGA2*), and chemokine signaling (*CCL5*), the SMAD family (*SMAD3*), the TCF family (*TCF12*). Further, as previously reported by Herndler-Brandstetter [6], we also saw alterations in DNA methylation of both *KLRG1* and *BACH2*, with the change in methylation of *KLRG1* occurring primarily in the comparison of CD8 central to effector memory and *BACH2* in central memory compared with naïve and effector memory. Like Rodriguez et al. [2], we observed substantial Ig-like (*KIR2DL4, KIR3DL3, KIRDP1*) and C-type lectin-like NK cell receptors (*KLRC3, KLRD1*), as well as mediators of NK cell activation (*VAV3, LYN*) genes with altered methylation comparing to CD8 TEMRA to effector cells.

Furthermore, 139 shared DMCs (out of 1000 compared), with 89 associated genes, were identified between CD8 TEMRA versus central and TEMRA versus effector memory comparisons. *BCL11B* appeared with the most CpG hits, showing 62 loci hypomethylated in TEMRA compared to central and effector cells. Studies have reported critical roles for *BCL11B* in T cell development and maintaining T cell identity [26, 27]. Peng et al. recently reported hydroxymethylation in the entire gene body of *BCL11B* in type 2 innate lymphoid cells, demonstrating innate lymphocyte epigenetic regulation by *BCL11B *[37]. Our methylation profiling of CD8 TEMRA is substantially consistent with previous research reporting the tight association between TEMRA transcriptional program and innate immunity phenotype. However, we also provided additional novel loci and genes like *BCL11B* specifically for TEMRA differentiation.

Examination of the data for B cell memory reveals that the data generated herein reflect the published literature. For example, as reviewed in Korosaki et al. [38], *PRDM1* and *BACH2* gene loci are directly involved in B cell memory differentiation. We find the levels of DNA methylation associated with both of these genes to differ between B naïve and memory cells. Further, the *AIM2* locus is critical for generating B cell immune memory [39], and we find clear differences in methylation at this locus. Like Kulis et al. [29], we find alterations in DNA methylation associated with the *BCL2* locus. Rodriguez-Cortez and colleagues [40] studied monozygotic twins discordant for immunodeficiency and reported numerous changes in DNA methylation in genes associated with B cell function. We observed many of the same genes as they report to have similar alterations, including the *BCL2* locus, numerous potassium channel regulators (*KCNAB1*, *KCNJ15*), *PTPRCAP*, *CCL5*, *RPTOR*, and *HDAC4.* At the same time, there are several additional genes that we have observed to have striking changes in DNA methylation that have not been previously tied to B cell memory generation. We identified the loss of methylation associated with the *SDK* gene to be prominent in B cell memory. This could be related to the changes in numbers and function of B memory cells in familial cases reported where germline deletions delete or truncate this gene [41]. Perhaps the most unusual of these is the *CAMTA1* gene, where we saw a significant decrease in methylation associated with the gene related to B cell memory. This gene has been implicated in plant immunity [42], biotic defense in Arabidopsis [43], and general stress and wound response in plants [44]. This gene is also required for long-term memory formation in the mouse [45]. Of course, it is unclear if this gene’s possible role in immune memory is related to neuronal memory, but the coincidence is intriguing.

We found 195 shared loci (out of 1000 compared), associated with 130 genes, to have altered methylation in B cell, CD4, and CD8 lineage during memory generation. They are considerably less methylated in memory cells than in naïve cells. These loci potentially provide information regarding the common pathways for memory lineage differentiation. The loci that we found to have the largest changes in DNA methylation in memory states compared to naïve states that are common to all three lineages code for proteins that participate in fundamental inflammatory processes. These methylation changes occurred in loci of genes associated with the inflammasome, interferons, stimulus and viral response networks, and stem-like pathways that feature microRNA regulation. The RANTES cytokine, coded for by the *CCL5* gene, has long been associated with T cell memory genesis (reviewed in Rahimi et al. [46]); its role in B cell memory is much less clear, but our data suggest further investigation is in order. *AIM2* is a dsDNA sensing protein that can initiate the formation of a distinct inflammasome. Svensson et al. [39] showed that *AIM2* is expressed in resting memory B cells but is down-modulated upon activation. Our data might suggest that this process is generalizable for all memory lineages. The *MITF* gene controls antigen expression and processing, including involvement (via interaction with DNA repair enzymes) with neoantigen formation [47, 48]. It is involved in B cell maturation, and our observations suggest that it also has a role in T cell memory. Indeed, the observation that the *RAD23A* gene (a DNA repair pathway member) is also considerably less methylated in memory cells is consistent with this, and RAD23 is also known to be a negative regulator of the anti-viral response [49]. The ATF/CREB family is well known to be a prominent signaling pathway in inflammation [50], with the *CREB5* gene methylation level having been shown to correlate with IL-6 levels [51]. Oddly, CREB-mediated transcription also enhances short- and long-term memory and is required for a stable recall of fear memory [52, 53]; it is unknown if this function is related to their role in immunological cell memory. As noted by Kim et al. [54] for T cell memory formation, microRNAs in general, and miR-21 in particular, play an important role. We have confirmed this, and our data further suggest that miR-21 also is important for B cell memory. LincRNAs are similar to microRNA, albeit they tend to target chromatin complexes and have a known role in immune cell homeostasis [55]. Several lincRNA-related loci were found to have altered methylation during memory activation in all three lineages, including *LINC01258* locus to be demethylated in the memory lineages. *LINC01258* is an ncRNA that targets the *PCGF5* polycomb complex, which has a key role in differentiation and the NOTCH signaling pathway [56]. *LINC01258* is upregulated in type 1 diabetes, a well-known immune disease [57]. Unlike naïve to memory activation, only 23 shared loci (out of 1000 compared) were identified for central to effector memory differentiation between CD4 and CD8 lineage, indicating unique epigenetic regulation pathways by lineage. Top genes identified for CD4 central to effector memory generation included *RBPJ*, a transcriptional regulator important in the Notch signaling pathway for CD4 T cell activation and differentiation [58, 59], and *CCNH*, which regulates cyclin-dependent kinase (CDK). Previous research showed that CDK inhibitors could regulate CD4 differentiation in response to mitogenic stimuli in mice [60]. For CD8 central to effector memory generation, top genes included JAK/STAT signaling pathway genes *JAK1* and *STAT4,* and antigen-presenting and membrane activity-related genes like *SCIMP, LAMC1, HLA-DPA1, KIAA0040, DOCK9,* and *TMEM63A.* Although limited shared top DMCs were observed for central to effector memory differentiation between CD4 and CD8 lineages, the biological processes enriched for both lineages are very similar, including stimulus and viral response, immune system process, and multiple cell level activities. The results suggest specific epigenetic regulation leading to common biological pathways for CD4 and CD8 central to effector memory activation.

Previous studies comparing DNA methylation in B and T cells found, almost exclusively, lineage-specific differential methylation that mapped to sites associated with combinations of transcription factor binding [61]. However, these studies did not consider the memory status of cells. Here, we identified 195 robust differences in methylation shared by CD4 T, CD8 T, and B memory cells compared to their naïve counterparts. These overlapping methylation sites were associated with a limited number of sequence motifs. For example, we observed enrichment for transcription binding sites of homeobox and POU transcription factors, including *POU6F1*, which was previously identified as being differentially expressed in murine and human memory cells [13]. The related *POU1F1* and *OTX1* factors are expressed in the developing brain, pituitary gland, and hematopoietic progenitors [62, 63]. Our analyses also highlight differential methylation of putative targets of *BACH2*, which is a highly conserved repressor that has been well described in T and B cell memory fate [64–66]. The nuclear hormone receptors *NR2F1* and *NR2F6* were enriched among overlapping memory methylation sites. The former is implicated in tumor cell dormancy [67], whereas the latter is implicated in the maintenance of peripheral immunological tolerance in T cells [68].

This study unveiled an epigenetically enriched genomic context for regulating memory generation. For all B cell, CD4, and CD8 memory activations, the DMCs are overrepresented in gene desert-like open sea and intergenic regions. This finding is consistent with previous research using DNA methylation to project peripheral immune cell proportions; immune cell type-specific CpGs were enriched in the open sea and underrepresented in CpG islands [69]. The results should promote further investigation of epigenetic regulation from gene desert and once-thought junk DNA on immune cellular identity and immunological processes.

While our study points to common epigenetic regulation during lymphocyte memory generation and establishes methylation patterns within memory cell differentiation, we recognize some limitations. First, although our research covers major naïve and memory cells in B cell, CD4, and CD8 lineages, lymphocytes are highly heterogeneous. We rely on widely accepted markers of these cells, although we note that adoptive transfer experiments required to prove memory capacity are not feasible in humans. Future studies addressing more cell subtypes are necessary to establish a more comprehensive epigenetic regulatory landscape of memory activation and differentiation. Second, although the methylation level alterations were found, their connection with gene expression change was only based on gene expression dataset enrichment and not on paired transcriptome analyses. Future analyses establishing methylation and gene expression change are necessary to understand better epigenetic and genetic regulatory networks in immune memory generation. Finally, more granular analyses on differentiating epigenetic pathways for memory cell differentiation are promised as we observed unique epigenetic changes leading to common biological processes.

## Conclusions

Our data suggest that epigenetic alterations are widespread and essential in generating human lymphocyte memory. Unique profiles are involved in methylation changes that accompany memory genesis in the three subtypes of lymphocytes. At the same time, several loci presumably play key overlapping roles in this memory process, where large methylation changes in the same gene locus are evident in all lymphocyte subtypes (e.g., *ATE1*, *AIM2*). Our exploration of the methylation profiles in memory cells has additional applications: it has made enhanced deconvolution of peripheral blood subtype profiles possible [69], allowing for more detailed immune system investigations using an epidemiologic approach. We believe it will be essential to explore the precise mechanisms these rich pathways represent, potentially providing novel insights into the similarity and difference in memory in different lymphocyte subtypes. Indeed, there are, no doubt, genetic mechanisms that impact these changes, and defining this extensive, rich methylation profile may further provide approaches to assess the role of genetics in the process. Finally, we also hope that explaining this normal process will rapidly expand and enhance our understanding of abnormal memory states’ genesis and provide new approaches to appreciate this as a basis for autoimmune and other diseases.

## Supplementary Information

**Additional file 1:** Supplementary Figures 1–3.

**Additional file 2:** Supplementary Table 1.

**Additional file 3:** Supplementary Table 2.

**Additional file 4:** Supplementary Table 3.

**Additional file 5:** Supplementary Table 4.

**Additional file 6:** Supplementary Table 5.

**Additional file 7:** Supplementary Table 6.

**Additional file 8:** Supplementary Table 7.

**Additional file 9:** Supplementary Table 8.

**Additional file 10:** Supplementary Table 9.

## Data Availability

All data sets used in this study are publicly available on Gene Expression Omnibus with the accession number GSE174666.

## Abbreviations

Bnv: B naïve cell
Bmem: B memory cell
CD4nv: CD4 naïve cell
CD4cm: CD4 central memory cell
CD4em: CD4 effector memory
CD8nv: CD8 naïve cell
CD8cm: CD8 central memory cell
CD8em: CD8 effector memory
TEMRA: CD8 terminally differentiated effector memory expresses CD45RA
DMCs: Differentially methylated CpGs
MSigDB: Molecular signature database
FDR: False discovery rate
