# Supplementary material for: Comparative analysis of the DNA methylation landscape in CD4, CD8, and B memory lineages
Source: Clin Epigenetics. 2022 Dec 15;14:173. doi: 10.1186/s13148-022-01399-0 (PMC9753273; doi:10.1186/s13148-022-01399-0)
Supplement: Supplementary file 1 — Additional file 1: Supplementary Figures 1–3. [file 13148_2022_1399_MOESM1_ESM.docx]

**
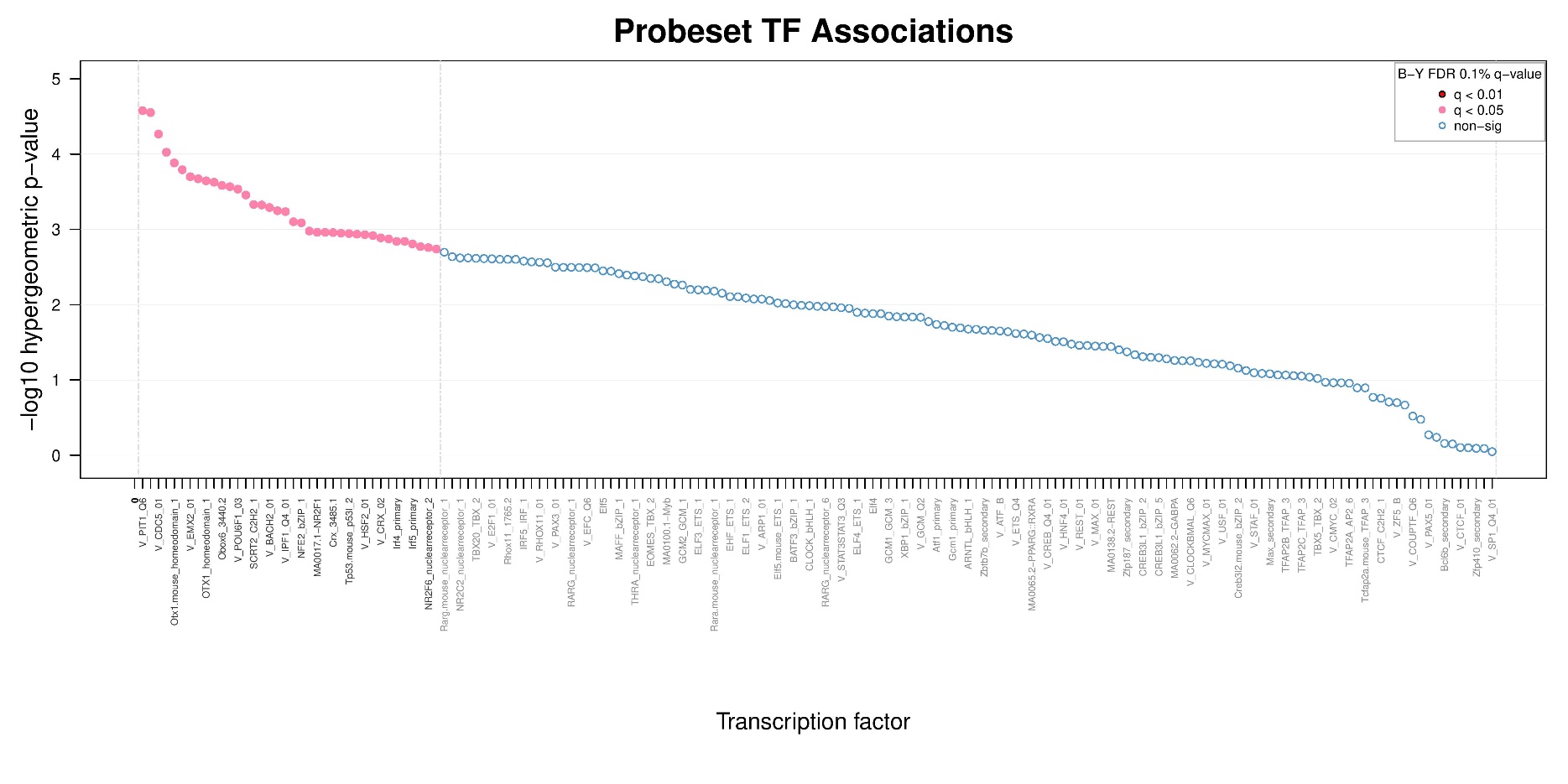
**

**Supplementary Figure 1.** Transcription factors enriched for B cell, CD4, and CD8 naive and memory differentially methylated loci.

**
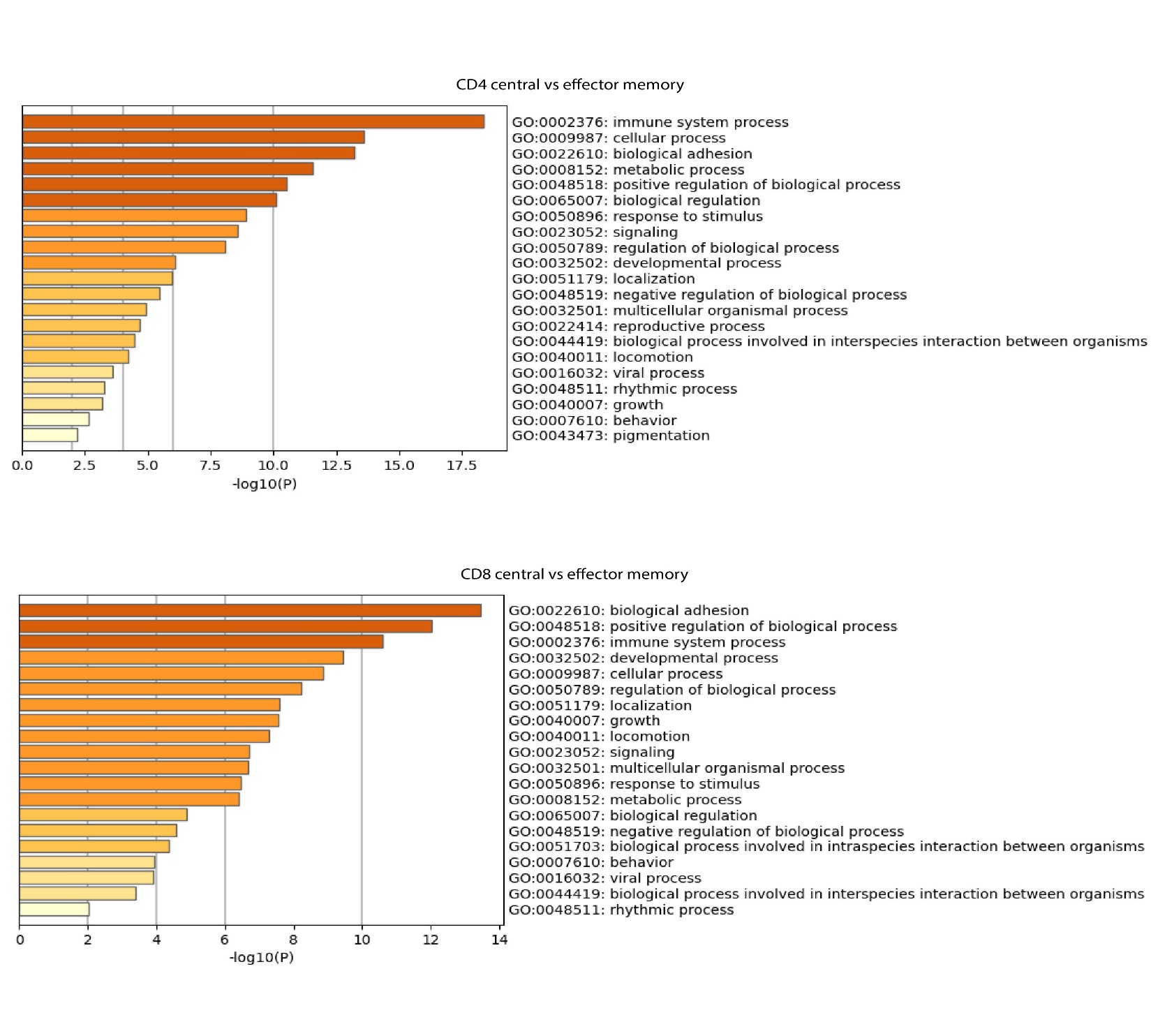
Supplementary Figure 2.** Gene ontology biological process enriched for CD4 and CD8 central and effector memory differentially methylated loci.


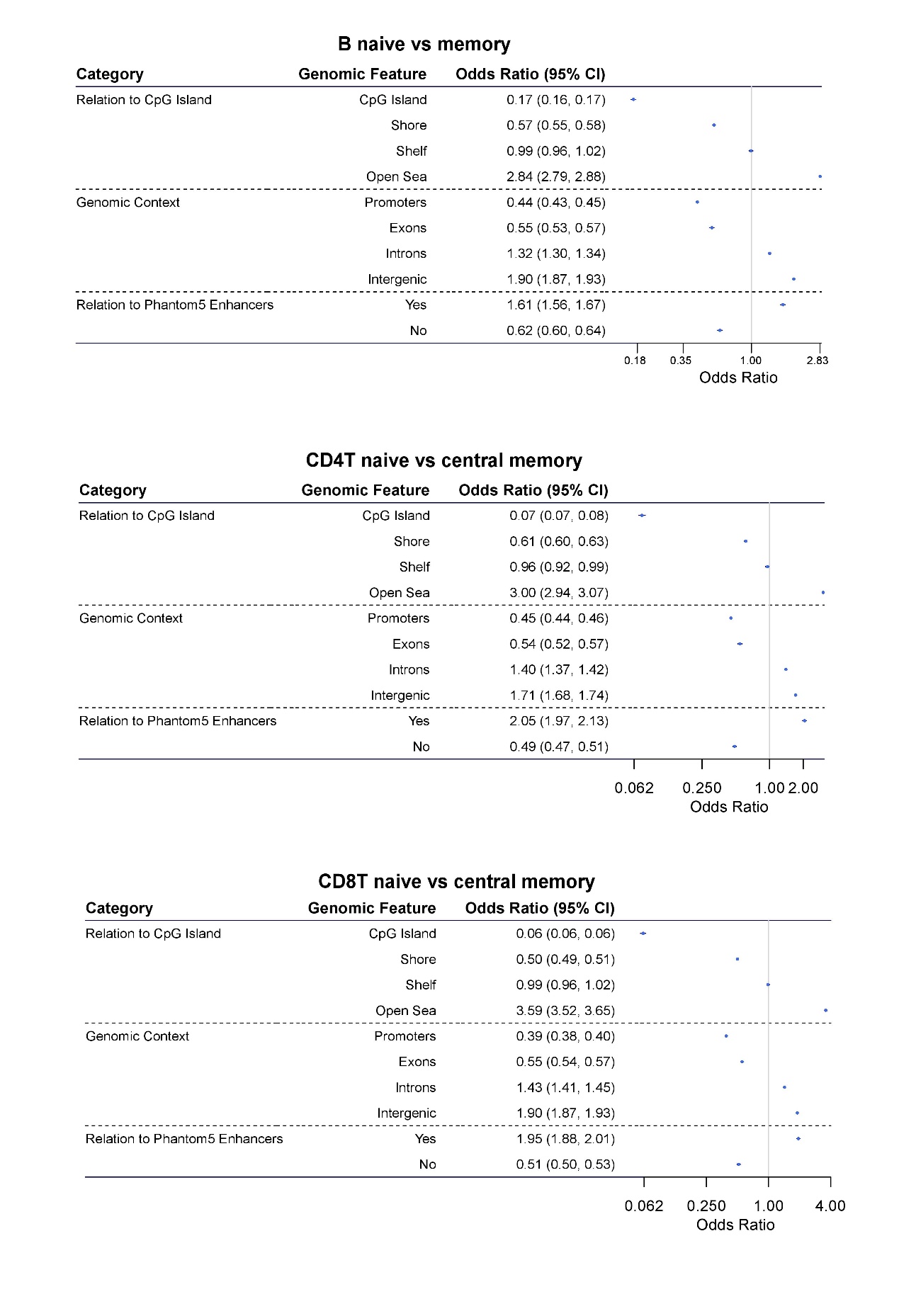
**Supplementary Figure 3.** Genomic context enriched for B cell, CD4, and CD8 naive and memory differentially methylated loci**.**

**
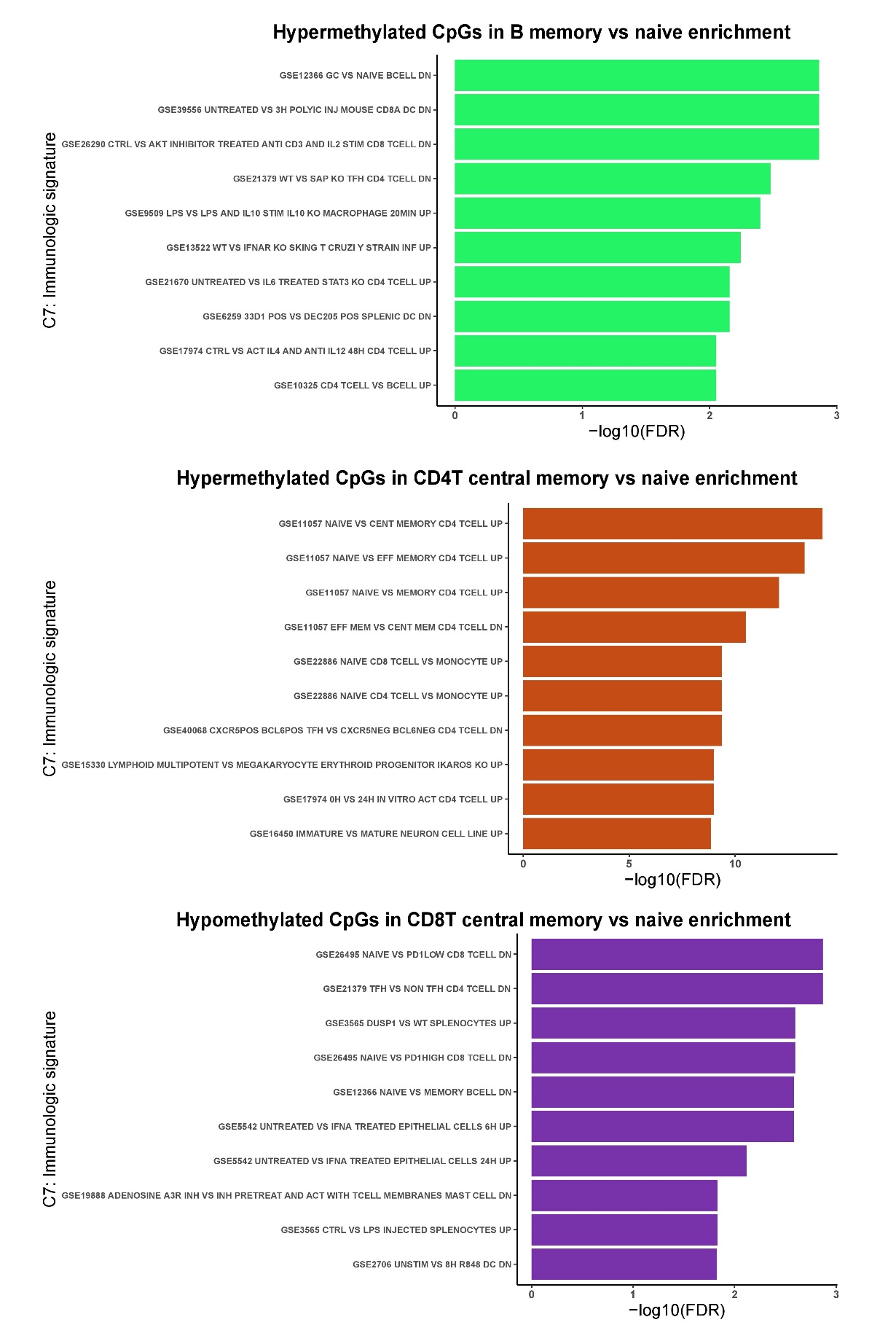
**

**Supplementary Figure 3.** C7: Immunological signatures enriched for B cell, CD4, and CD8 naive and memory differentially methylated loci**.**
